# O.4.5-8 Intergenerational transmission of sport club participation

**DOI:** 10.1093/eurpub/ckad133.216

**Published:** 2023-09-11

**Authors:** Kasper Salin

**Affiliations:** University of Jyväskylä, Finland; kasper.salin@jyu.fi

## Abstract

**Purpose:**

The Purpose of the study was to investigate the association of parents physical activity (PA) to the offspring PA in different ages. The main research question was to find out whether there is intergenerational transmission of sport club participation and how this is related to the gender of parent.

**Methods:**

The study included data from two generations, G0 (parents) and G1 (offspring). G0 included parents from six cohorts, aged 41-56, a total of 2,324 (54% female). For the sport participation analyses, children and adolescents aged 9-18 were included, consisting of 1269 participants (52% female). Self-reported questionnaire of parents PA was collected in 1980-1989 and similar questions were collected from offspring in 2018-2020.

**Results:**

Parents sport club participation in childhood at age 9 was associated with offspring participation in ages 9-15 (.315-223**), at age 12 with ages 9-12 (.240**-.135*), at age 15 only with age 12 (.180*) and at age 18 with ages 12-15 (.225-176*). Girls (offspring) participation in younger ages seemed to be associated with parents’ participation in ages 9-15 (.385*-.326), while among boys, participation in age 18 was associated with parents participation in ages 9-12;18, (.564-390*). Association between mother and son was found especially in ages 9-12 (.275-.441*), while father’s participation in age 18 was associated with sons’ participation at the age of 18 (.634**) and with daughter’s participation at the age of 15 (.377*).

**Conclusions:**

Parent’s example seems to be important at the younger ages to engage to sport participation. Mothers and fathers have different influence on PA of their offspring.

**Support/Funding Source:**

The work was supported by the Ministry of Education and Culture (36/626/2020).

